# Treatment of Myeloproliferative Neoplasms With Janus Kinase Inhibitors: A Meta‐Analysis of Cardiovascular Safety

**DOI:** 10.1002/jha2.70000

**Published:** 2025-02-12

**Authors:** Roberta Dunn, Edouard Long, Laura Li Gagnon, Claire Harrison, Yunfan Yang, Jennifer O'Sullivan

**Affiliations:** ^1^ GKT School of Medical Education King's College London London UK; ^2^ Department of Haematology Guy's and St. Thomas' NHS Foundation Trust London UK; ^3^ Department of Haematology CHU de Québec‐Université Laval Quebec Canada; ^4^ Department of Hematology West China Hospital of Sichuan University Chengdu Sichuan China

**Keywords:** cardiology, meta‐analysis, myeloproliferative disease, myeloproliferative disorder, thrombosis

## Abstract

**Background:**

Janus kinase inhibitors (JAKis) are an integral aspect of the management of myeloproliferative neoplasms (MPNs). Part of the clinical benefit derived from JAKis may be due to reductions in thrombosis, a potentially life‐threatening complication of MPNs. However, evidence has emerged of adverse cardiovascular effects secondary to JAKis. We conducted a first‐of‐a‐kind meta‐analysis of the cardiovascular safety of JAKis in the treatment of MPNs.

**Methods:**

This study was conducted according to the Preferred Reporting Items for Systematic Reviews and Meta‐Analysis (PRISMA). Systematic searches for studies comparing JAKi treatment to a control group were conducted. Studies reporting hypertension, major adverse cardiovascular events (MACE) and thromboembolic events were included in a meta‐analysis using a random‐effects model for the primary analysis, and fixed‐effects model for any subgroup analyses performed.

**Results:**

A total of 23 publications, consisting of nine clinical trials and one retrospective analysis, met the inclusion criteria. This resulted in a pooled population of 2198 patients (JAKi *n* = 1145, Control *n* = 1053). In studies reporting thromboembolic events (*n* = 9), pooled analysis revealed a significantly lower rate of thromboembolic events in the JAKi group (incidence rate ratio (IRR): 0.52, 95% CI: 0.28–0.98, *p* = 0.04). This was primarily driven by ruxolitinib studies in myelofibrosis (MF) and polycythemia vera (PV) as when a subgroup analysis of these trials was performed (*n* = 7), an even more significant reduction in thromboembolic events with JAKi treatment was found (IRR: 0.41, 95%CI: 0.26–0.64, *p* < 0.001). There was no significant difference in MACE or hypertension between JAKi and control groups.

**Conclusion:**

This meta‐analysis suggests that JAKi treatment of MPN was associated with a reduced risk of thromboembolic events; primarily driven by studies of ruxolitinib in PV and MF. Further prospective clinical trials are warranted to confirm these findings and characterise the cardiovascular profile of other JAKis and other types of MPNs.

## 1 Introduction

Myeloproliferative neoplasms (MPNs)—the main subtypes of which are polycythemia vera (PV), essential thrombocythemia (ET) and myelofibrosis (MF)—are a group of haematological malignancies characterised by the constitutive activation of the Janus kinase (JAK) / signal transducer and transcription activator protein (STAT) pathway in clonal myeloid precursors. [1].

JAK inhibitors (JAKis), first approved by the Food and Drug Administration (FDA) in 2011 for the treatment of adults with MF, are a cornerstone of the management of MPNs. Of the four JAKis which have completed phase three trials—ruxolitinib, pacritinib, fedratinib and momelotinib—large studies such as COMFORT, MAJIC‐PV, PERSIST and MOMENTUM have demonstrated these medications to be effective in improving outcomes in MF and PV, with mixed results in ET [2, 3, 4, 5]. However, there have been observed increases in body weight and cholesterol levels [6] secondary to JAKis, both key risk factors for cardiovascular disease [7]. Moreover, the PERSIST‐2 clinical trial was terminated prematurely due to concerns over interim survival results showing increases in bleeding and cardiovascular events in the JAKi arm [8].

Previously, the primary endpoint for clinical trials in MF and PV has been control of symptoms, spleen size and haematocrit. However, thrombosis is a relatively common and potentially life‐threatening complication associated with MPNs [9]. Hence, it is important to understand the impact of JAKi treatment on thrombotic risk. A meta‐analysis including 750 patients by Samuelson et al. found rates of thrombosis were significantly lower among patients treated with ruxolitinib (risk ratio (RR) 0.45, 95% CI 0.23–0.88) compared to control [10].

There is evidence that the use of JAKis may increase the rate of major adverse cardiovascular events (MACEs)—a composite measure of cardiovascular adverse events frequently used in clinical trials. The ORAL Surveillance trial investigating the safety of the JAKi tofacitinib for the treatment of rheumatoid arthritis (RA) [11] concluded that JAKi treatment was associated with an increased risk of MACE and did not meet non‐inferiority criteria, leading to the addition of a boxed warning on all JAKis used for the treatment of inflammatory conditions. This safety analysis of JAKi in RA was prompted by several studies [12, 13] illustrating that JAKi treatment in RA was associated with increases in cholesterol and lipid levels. Moreover, a large meta‐analysis of 126,961 patients with immune‐mediated inflammatory disorders found JAKi treatment to be associated with a higher risk of MACE compared to control [14].

Here we report a first‐of‐a‐kind meta‐analysis of the cardiovascular safety of JAKis in the treatment of MPNs. We aim to compare the rates of both arterial and venous thrombosis, MACE and hypertension [7]—another key risk factor for cardiovascular disease—in MPN patients treated with JAKi compared to those treated with best available therapy (BAT), or placebo, and comment on the clinical significance of cardiovascular safety in this group of patients.

## 2 Methods

### 2.1 Search Strategy

This systematic review and meta‐analysis were carried out in accordance with the Preferred Reporting Items for Systematic Reviews and Meta‐Analyses (PRISMA) [15]. Ethical approval from an institutional review board was not required as this is a retrospective trial‐level meta‐analysis. Systematic searches were conducted in PubMed, Ovid Embase, Ovid MEDLINE and the Cochrane Library from inception to February 1, 2024. Medical Education Subject Headings and key terms were combined with Boolean operators to retrieve publications investigating the treatment of MPNs with JAKis that reported cardiovascular adverse events (AEs) included in the primary outcomes. Publicly available data from clinicaltrials.gov was used to supplement AE event rates reported in publications.

### 2.2 Inclusion and Exclusion Criteria

We included clinical trials and cohort studies, both prospective and retrospective, which compared a JAKi to a control group which did not include a JAKi. Single‐arm studies, conference reports and systematic reviews were excluded. To meet the inclusion criteria, any outcome of MACE, thrombosis or hypertension had to be reported in either the full manuscript, its corresponding supplementary material or on clinicaltrials.gov.

### 2.3 Outcomes

The primary outcomes were the incidence of (1) thromboembolic events, (2) MACE or (3) hypertension. MACE was defined as a composite of cerebrovascular events, myocardial infarction (MI), cardiac arrest and heart failure. If no overall figure was reported for thromboembolic events then a composite of MI, deep vein thrombosis (DVT), pulmonary embolism (PE), portal vein thrombosis, splenic infarction, thrombophlebitis and thrombosis was calculated. In studies where only certain components of MACE or thromboembolic events were reported, the composite was calculated using the available adverse event data reported in manuscripts, Supporting Information and clinicaltrials.gov. These can be found in Tables S1 and S2.

### 2.4 Study Selection and Extraction

Two reviewers (R.D. and E.L.) independently screened titles and abstracts for eligibility and discrepancies were resolved by consensus. Publications were then screened for final selection based on the selection criteria. Where several publications reported on the same study population, only data from the most recent record which reported adverse events of interest was included. Expert opinion (L.L.G, C.H., Y.Y and J.O.) was then sought to identify extra studies for inclusion. In cases where crossover was allowed, the crossover group was not included in the cumulative event rate calculation to mitigate the impact of recent JAKi treatment on our results. Data were then extracted and collated into an electronic database. The methodological quality of the included trials was evaluated using the Cochrane Risk‐of‐bias Tool 2 [13] for randomised controlled trials and the ROBINS‐I [14] checklist for non‐randomised study designs.

### 2.5 Statistical Analysis

For each primary outcome, incidence rates were calculated as event rate per 100 patient‐years. This was calculated by dividing the total number of events by the total patient exposure time, in 100y units. In cases where patient‐year exposure time was not reported, this was calculated by multiplying the number of patients with the median length of follow‐up for a given arm.

Incidence rate ratios (IRRs) and 95% confidence intervals (CIs) were pooled using a random effects model, with heterogeneity assessed using *I* ^2^ and Cochran's *Q* indices. A random‐effects model was used in favour of a fixed‐effects model for the primary analysis, due to the heterogeneity of MPNs and JAKis in the included studies. Subgroup analyses of the same JAKi were pooled with a fixed‐effects model. Heterogeneity was categorised as low (*I* ^2^ < 30%), moderate (*I* ^2^ = 30%–60%) or high (*I* ^2^ > 60%); significant moderate heterogeneity (*p* < 0.05) prompted sensitivity analyses by excluding individual studies to identify sources of heterogeneity. Statistical significance was defined as *p* < 0.05. All statistical analyses were performed using R version 4.4.0 (R Foundation for Statistical Computing, Vienna, Austria).

## 3 Results

**FIGURE 1:**
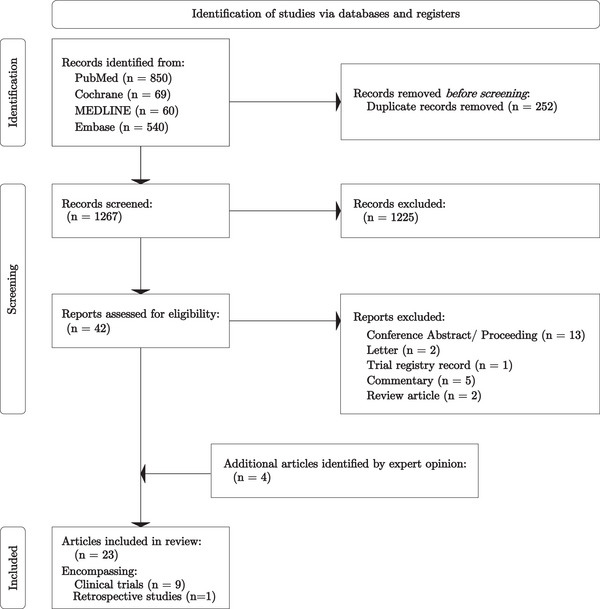
PRISMA flow diagram.

Using the search strategy described above, 1519 publications were identified (Pubmed = 850, Cochrane Library = 69, MEDLINE = 60, Embase = 540). A total of 252 papers were duplicates which were removed before screening. Following screening of the title and abstract against the pre‐defined selection criteria, 1225 further publications were excluded. A full‐text review of 42 publications, alongside 4 additional publications identified through expert opinion, was undertaken yielding 23 publications meeting the inclusion criteria. After taking the most recent publication for manuscripts which reported on the same population of individuals, nine clinical trials and one retrospective analysis were included in the meta‐analysis (Figure 1). Risk‐of‐bias analysis was conducted for all ten studies, revealing nine studies with low bias and one study with moderate bias (Figures S1 and S2). It is worth noting that numerous large trials investigating JAKis in MPNs did not meet the inclusion criteria: the control group included JAKi in PERSIST‐1, SIMPLIFY‐1, SIMPLIFY‐2 and FREEDOM2; JAKARTA2 and FREEDOM were single‐arm studies; and JAKARTA did not publicly report any primary outcome data [16].

### 3.1 Study and Baseline Characteristics

Study and baseline characteristics are presented in Table 1. The studies enrolled a total of 2198 patients (JAKi *n* = 1145, Control *n* = 1053) to three JAKis: ruxolitinib (*n* = 8), pacritinib (*n* = 1) and momelotinib (*n* = 1). The MPNs included were MF (4 studies), ET (1 study) and PV (5 studies). Median age was similar for the two cohorts (JAKi: 66 years, control: 67 years) and the control cohort had a slightly higher proportion of male patients (JAKi: 53%, Control: 58%). Nine studies reported data on thromboembolic events, six studies reported data on hypertension and all studies reported data on MACE.

**TABLE 1:** Study and baseline characteristics.

First author, year published	Study name	MPN	JAKi	Comparator^a^	Median age (y)		Male (%)		Patients (*n*)		Length of follow‐up (y)		Thromboembolic events	Hypertension	MACE
					JAKi	Control	JAKi	Control	JAKi	Control	JAKi	Control			
Verstovsek, 2017 [2]	COMFORT‐I	MF	Ruxolitinib	Placebo	66	70	51	57	155	151	2.86	0.71	Y	–	Y
Harrison, 2016 [22]	COMFORT‐II	MF	Ruxolitinib	BAT	67	66	57	58	146	73	4.7	2.9	Y	Y	Y
Harrison, 2017 [5]	MAJIC‐ET	ET	Ruxolitinib	BAT	63	66	38	42	58	57	2.61	2.61	Y	–	Y
Mesa, 2017 [23]	RELIEF	PV	Ruxolitinib	HC	64	66	44	61	54	56	1	1	Y	Y	Y
Mesa, 2017 [4]	PERSIST‐1	MF	Pacritinib	BAT	67	65	57	56	220	106	1.93	1.95	–	Y	Y
Kiladjian, 2020 [24]	RESPONSE	PV	Ruxolitinib	BAT	62	60	60	71	110	111	3.89	0.66	Y	Y	Y
Passamonti, 2022 [25]	RESPONSE‐2	PV	Ruxolitinib	BAT	63	67	53	63	74	75	4.46	0.71	Y	Y	Y
Alvarez‐Larrán, 2022 [26]	–	PV	Ruxolitinib	BAT	71	74	53	49	105	272	2.39	4.68	Y	–	Y
Verstovsek, 2023 [20]	MOMENTUM	MF	Momelotinib	Danazol	71	72	61	68	130	65	0.30	0.51	Y	Y	Y
Harrison, 2023 [27]	MAJIC‐PV	PV	Ruxolitinib	BAT	67	66	60	56	93	87	4.8	4.8	Y	–	Y

*Note*: ‘–’ means not reported.

Abbreviations: BAT: Best available therapy, ET: Essential thrombocytosis, HC: hydroxycarbamide, JAKi: Janus kinase inhibitor, MACE: Major cardiovascular adverse events, MF: Myelofibrosis, MPN: Myeloproliferative neoplasm, PV: Polycythemia vera.

^a^ For a detailed breakdown of comparators, please see Table S3.

### 3.2 Thromboembolic Events

Nine studies with a total of 1872 patients were included in the thrombosis analysis. A total of 950 patients received JAKis and 922 patients were in the control group. Studies included ruxolitinib (*n* = 8) and momelotinib (*n* = 1) in patients with PV (*n* = 5), MF (*n* = 3) and ET (*n* = 1). Average follow‐up was 3.08 years in the JAKi group and 1.74 years in the control group.

**FIGURE 2:**
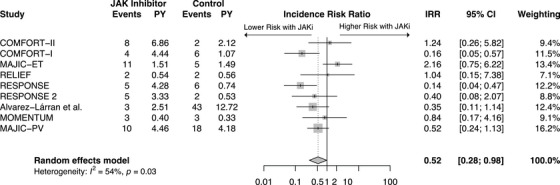
Forest plot of all thromboembolic events comparing JAK inhibitor to control. JAK inhibitor—Janus kinase inhibitor, IRR—Incidence rate ratio, CI—Confidence interval, PY—Patient years at risk (in 100y).

**FIGURE 3:**
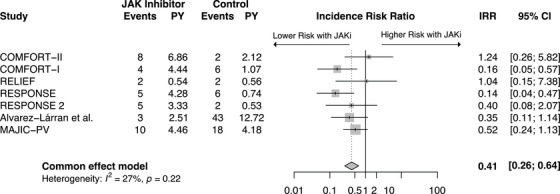
Forest plot of all thromboembolic events subgroup analysis comparing ruxolitinib to control in MF and PV. MF—Myelofibrosis, PV—Polycythemia vera, JAK inhibitor—Janus kinase inhibitor, IRR—Incidence rate ratio, CI—Confidence interval, PY—Patient years at risk (in 100y).

In pooled analysis (Figure 2), there were significantly fewer thromboembolic events in the JAKi group compared to control (IRR: 0.52, 95% CI: 0.28‐0.98, *p* = 0.04) (*I* ^2^ = 53.9%). This finding was primarily driven by ruxolitinib studies in MF and PV. When a subgroup analysis of these studies (*n* = 7) was performed, an even more significant reduction in thromboembolic events with JAKi treatment was found (IRR: 0.41, 95%CI: 0.26‐0.64 *p* < 0.001) (*I* ^2^ = 27.4%) (Figure 3). Although the pooled analysis of all JAKis demonstrated high heterogeneity (*I* ^2^ = 53.9%, *p* < 0.05), removal of MAJIC‐ET and MOMENTUM in the subgroup analysis drastically reduced the heterogeneity (*I* ^2^ = 27.4%, *p* > 0.05).

### 3.3 MACE

**FIGURE 4:**
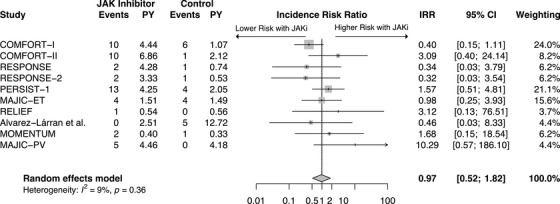
Forest plot of MACE comparing JAK inhibitor to control. MACE—Major adverse cardiovascular events, JAK inhibitor—Janus kinase inhibitor, IRR—Incidence rate ratio, CI—Confidence interval, PY—Patient years at risk (in 100y).

Ten studies with a total of 2198 patients, 1145 in the JAKi group and 1053 in the control group, were included in the MACE analysis. Studies encompassed ruxolitinib (*n* = 8), momelotinib (*n* = 1) and pacritinib (*n* = 1) in patients with PV (*n* = 5), MF (*n* = 4) and ET (*n* = 1). There was an average follow‐up of 2.89 years in the JAKi group and 2.05 years in the control group. In pooled analysis (Figure 4), there was no significant difference in the rate of MACE in patients treated with JAKi compared to control (IRR: 0.97, 95% CI: 0.52–1.82, *p* = 0.93) (Figure 4). This analysis had a low heterogeneity (*I* ^2^  = 8.8%), so no additional analysis was necessary.

### 3.4 Hypertension

Six studies were included in the analysis of hypertension. A total of 734 patients received JAKi treatment and 486 patients received control treatment. Ruxolitinib (*n* = 4), pacritinib (*n* = 1) and momelotinib (*n* = 1) were all included in the analysis. There was an equal number of studies for MF (*n* = 3) and PV (*n* = 3). The JAKi and control group had an average follow‐up period of 2.61 and 1.14 years, respectively.

**FIGURE 5:**
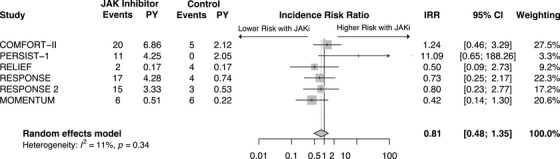
Forest plot of hypertension comparing JAK inhibitor to control. JAK inhibitor—Janus kinase inhibitor, IRR—Incidence rate ratio, CI—Confidence interval, PY—Patient years at risk (in 100y).

There was no significant difference in hypertension associated with JAKis compared to control (IRR 0.81, 95%CI: 0.48‐1.35, *p* = 0.42) (Figure 5). This analysis had low heterogeneity (*I* ^2^ = 11.3%), so no additional analysis was necessary.

## 4 Discussion

In this meta‐analysis of the cardiovascular safety of JAKis in the treatment of MPNs including over 2000 patients over ten studies, our key findings are threefold: (1) JAKi treatment is associated with a significant reduction in thromboembolic event risk, primarily driven by studies of ruxolitinib in MF and PV. (2) JAKi treatment is not associated with an increase in MACE in patients with MPNs. (3) The risk of hypertension is not significantly increased by JAKi treatment in patients with MPNs.

In a recent review of cardiovascular disease in MPNs, it has been postulated that there are four possible mechanisms for an increased propensity of thromboembolic events and cardiovascular disease more generally [6]. Inherent to MPNs, their effect on the numbers and function of numerous blood cells, including red blood cells and platelets, can interact and activate the coagulation pathway contributing to the risk of thrombosis. Secondly, the activation of the JAK/STAT signalling pathway at the centre of the pathogenesis of MPNs promotes the expression of inflammatory cytokines, such as IL‐6 and tumour necrosis factor‐alpha, which may themselves promote atherogenesis. Furthermore, the activation of these cytokines can lead to vascular injury and a pro‐thrombotic state due to their harmful effect on vascular endothelial cells [9]. Thirdly, increases in tumour growth factor beta and the enzyme lysyl oxidase expression by MPNs may further contribute to atherogenesis and thrombosis. Finally, there is evidence that increased activation of the renin‐angiotensin system by the overactivity of the JAK/STAT pathway may cause deleterious effects on the cardiovascular system.

In the context of MPNs, our finding that JAKi treatment reduces the risk of thrombosis has been observed by others including Samuelson et al. who demonstrated that rates of thrombosis were significantly lower in patients treated with ruxolitinib (IRR: 0.45, 95% CI: 0.23–0.88) [10]. In terms of cardiovascular safety more generally, a 10‐year review of the safety of ruxolitinib concluded that there was no indication of ruxolitinib treatment in patients with MF or PV increased the risk of MACE [6]. Although the cardiovascular risk profile of pacritinib has been flagged previously, a recent risk‐adjusted safety analysis of the PERSIST trials, which took into account the differential time of treatment of the BAT arm compared to the pacritinib arm, concluded that there was no excess in bleeding, cardiac events or thrombosis in patients treated with pacritinib [17].

It should also be noted that the cardiovascular safety of JAKis in other patient populations has been investigated. In the treatment of atopic dermatitis, JAKi treatment posed little‐to‐no effect on the risk of MACE [18]. These findings were corroborated in a large cohort of patients with immune‐mediated inflammatory skin diseases [19].

In recent post hoc analyses of the original ORAL Surveillance study in RA, evidence is emerging that the increased rates of MACE secondary to JAKi treatment primarily occurred in patients who had pre‐existing atherosclerotic cardiovascular disease [20]. This suggests that the cardiovascular risk profile of JAKi treatment in MPNs may be different in patients with pre‐existing cardiovascular co‐morbidities. This supports an individualised approach to cardiovascular risk management in MPN patients, notably since they are already at increased risk of cardiovascular complications. Although both rheumatoid disease and MPNs exhibit a chronic inflammatory state, differences underlying the mechanism and management of these diseases may be responsible for their differing cardiovascular risk profiles. As aspirin is routinely used in the treatment of MPNs to reduce the risk of thromboembolic events in the microvasculature [21], it is plausible this may account for the difference in MACE in patients with RA and MPNs.

Although this analysis found no significant increased adverse risk of hypertension and MACE with JAKi treatment, JAKi are known to impact cholesterol and cause weight gain [6, 7] and so cardiovascular health should continue to be closely monitored in this patient population.

### 4.1 Study Limitations

There are several limitations to this work which must be acknowledged. We only had access to publicly available data so our calculation of event rates may differ from the true rate, especially since certain studies did not report categories of interest in our primary outcomes. Moreover, our thromboembolic event and MACE definitions were not adjudicated by a central committee and so may be heterogenous between trials. There was also a large variation in follow‐up duration between JAKi and control groups and between the trials themselves. As noted in our results, the majority of trials included were for ruxolitinib so these results may not be generalisable to all JAKis used in MPN treatments. This was mainly due to the inclusion of JAKi, usually ruxolitinib, in the control group of more recent JAKi trials in MPNs. Most of the included studies were clinical trials which may disproportionately exclude individuals with significant comorbidities, introducing a degree of bias which may limit the generalisability of our results to the wider population. Finally, the heterogeneity of MPNs, JAKis and controls used within the included trials may have introduced confounding factors, limiting the generalisability of our results. In particular, the heterogenous cytoreductive drugs used as control may each have separate cardiovascular risk profiles which could have influenced the risk of complications and different MPN subtypes may predispose patients to varying levels of risk of adverse cardiac complications.

## 5 Conclusion

In this meta‐analysis, JAKi treatment for the treatment of MPNs was associated with a reduced risk of thromboembolic events compared to control, primarily driven by studies of ruxolitinib in PV and MF. JAKi treatment was not associated with an increased risk of MACE or hypertension, adding to the existing body of evidence demonstrating the safety of JAKi in the treatment of MPNs. Further prospective clinical trials are warranted to confirm these findings and characterise the cardiovascular profile of other JAKis in all types of MPNs.

## Author Contributions

Conceptualisation: Roberta Dunn, Claire Harrison, Yunfan Yang and Jennifer O'Sullivan. Data curation: Roberta Dunn and Edouard Long. Formal analysis: Roberta Dunn and Edouard Long. Investigation: Roberta Dunn and Edouard Long. Methodology: Roberta Dunn and Edouard Long. Visualisation: Roberta Dunn and Edouard Long. Writing – original draft: Roberta Dunn and Edouard Long. Writing – review and editing: all authors. Supervision: Laura Li Gagnon, Claire Harrison, Yunfan Yang and Jennifer O'Sullivan.

## Ethics Statement

The authors have nothing to report.

## Consent

The authors have nothing to report.

## Conflicts of Interest

Claire N. Harrison has received institutional grants from Constellation and Novartis; consultation fees from Novartis, MSD, Karyopharm, AOP, GlaxoSmithKline, BMS, Sobi, Galecto and CTI; honoraria fees from Novartis, MSD, Karyopharm, Sobi, GlaxoSmithKline and BMS; support for attending meetings and/or travel from Novartis; participated on a Data Safety Monitoring Board or Advisory Board for BMS and Galecto; leadership role with Blood Cancer UK (Trustee; unpaid), EHA (Deputy Editor‐in‐Chief remunerated) and MPN Voice (Medical Director; unpaid); and holds stock or stock options with Chakana Medical Limited.

## Clinical Trial Registration

The authors have confirmed clinical trial registration is not needed for this submission.

## Supporting information



Supporting Information

## Data Availability

The data underlying this article will be shared on reasonable request to the corresponding author.
