# Supplementary material for: Treatment of Myeloproliferative Neoplasms With Janus Kinase Inhibitors: A Meta‐Analysis of Cardiovascular Safety
Source: EJHaem. 2025 Feb 12;6(1):e70000. doi: 10.1002/jha2.70000 (PMC11815325; doi:10.1002/jha2.70000)
Supplement: Supplementary file 1 — Supporting Information [file JHA2-6-e70000-s001.docx]

**SUPPLEMENTARY MATERIAL**

**Supplementary Table S1: Calculation of all thromboembolic events composite**

| First Author, Year Published | Study Name | MI (n) | | DVT (n) | | PE (n) | | Portal Vein Thrombosis (n) | | Splenic Infarction (n) | | Thrombophlebitis (n) | | Thrombosis (n) | | All Thromboembolic Events (n) | | All Thromboembolic Events (/100PY) | |
| --- | --- | --- | --- | --- | --- | --- | --- | --- | --- | --- | --- | --- | --- | --- | --- | --- | --- | --- | --- |
|  |  | JAKi | Control | JAKi | Control | JAKi | Control | JAKi | Control | JAKi | Control | JAKi | Control | JAKi | Control | JAKi | Control | JAKi | Control |
| Verstovsek,  2017 | COMFORT-I | 3 | 1 | - | - | 0 | 1 | - | - | 1 | 4 | - | - | - | - | 4 | 6 | 0.90 | 5.61 |
| Harrison,  2016 | COMFORT-II | 2 | 0 | 2 | 1 | 1 | 0 | 2 | 1 | 1 | 0 | - | - | 0 | 0 | 8 | 2 | 1.7 | 0.94 |
| Harrison,  2017 | MAJIC-ET | *Composite figure reported | | | | | | | | | | | | | | 11 | 5 | 7.27 | 3.36 |
| Mesa,  2017 | RELIEF | *Composite figure reported | | | | | | | | | | | | | | 2 | 2 | 3.71 | 3.57 |
| Kiladjian**,**  2020 | RESPONSE | *Composite figure reported | | | | | | | | | | | | | | 5 | 6 | 1.2 | 8.2 |
| Passamonti,  2022 | RESPONSE-2 | *Composite figure reported | | | | | | | | | | | | | | 5 | 2 | 1.5 | 3.7 |
| Alvarez-Larrán,  2022 | - | *Composite figure reported | | | | | | | | | | | | | | 3 | 43 | 1.20 | 3.38 |
| Verstovsek,  2023 | MOMENTUM | 1 | 0 | - | - | 1 | 1 | - | - | 1 | 2 | - | - | - | - | 3 | 3 | 7.59 | 9.04 |
| Harrison, 2023 | MAJIC-PV | *Composite figure reported | | | | | | | | | | | | | | 10 | 18 | 2.24 | 4.31 |

*In instances where studies reported the number of instances of ‘all thromboembolic events’, this was used over calculating a composite of each individual category of thromboembolic event

‘ – ‘ means not reported

Abbreviations: MI: myocardial infarction, DVT: deep vein thrombosis, PE: pulmonary embolism, /100PY: per 100 patient-years, JAKi: Janus kinase inhibitor

**Supplementary Table S2: Calculation of MACE composite**

| First Author, Year Published | Study Name | Cerebrovascular event (n) | | MI (n) | | Cardiac arrest (n) | | Cardiac/heart failure (n) | | MACE (n) | | MACE (/100PY) | |
| --- | --- | --- | --- | --- | --- | --- | --- | --- | --- | --- | --- | --- | --- |
|  |  | JAKi | Control | JAKi | Control | JAKi | Control | JAKi | Control | JAKi | Control | JAKi | Control |
| Verstovsek,  2017 | COMFORT-I | 2 | 1 | 3 | 1 | 1 | 0 | 4 | 4 | 10 | 6 | 2.25 | 5.61 |
| Harrison,  2016 | COMFORT-II | 3 | 0 | 1 | 0 | 1 | 0 | 5 | 1 | 10 | 1 | 1.46 | 0.47 |
| Harrison,  2017 | MAJIC-ET | 2 | 4 | 2 | 0 | - | - | - |  | 4 | 4 | 2.64 | 2.69 |
| Mesa,  2017 | RELIEF | 1 | 0 | - | - | - | - | - | - | 1 | 0 | 1.86 | 0.00 |
| Mesa,  2017 | PERSIST-1 | 1 | 0 | - | - | 1 | 0 | 11 | 4 | 13 | 4 | 3.06 | 1.95 |
| Kiladjian**,**  2020 | RESPONSE | 1 | 0 | 0 | 1 | - | - | 1 | 0 | 2 | 1 | 0.47 | 1.36 |
| Passamonti,  2022 | RESPONSE-2 | 1 | 0 | 1 | 0 | - | - | 0 | 1 | 2 | 1 | 0.60 | 1.87 |
| Alvarez-Larrán,  2022 | - | - | - | - | - | - | - | 0 | 5 | 0 | 5 | 0.00 | 0.39 |
| Verstovsek,  2023 | MOMENTUM | 1 | 1 | 1 | 0 | 0 | 0 | 0 | 0 | 2 | 1 | 5.06 | 3.01 |
| Harrison, 2023 | MAJIC-PV | 4 | 0 | - | - | - | - | 1 | 0 | 5 | 0 | 1.12 | 0.00 |

‘ – ‘ means not reported

Abbreviations: MACE: Major adverse cardiovascular event, MI: myocardial infarction, /100PY: per 100 patient-years, JAKi: Janus kinase inhibitor

**Supplementary Table S3: Detailed Breakdown of Study Comparators**

| First Author, Year Published | Study Name | Comparator | Change of BAT therapy permitted |
| --- | --- | --- | --- |
| Verstovsek,  2017 | COMFORT-I | Placebo | N/A |
| Harrison,  2016 | COMFORT-II | ‘Any commercially available agents (as monotherapy or in combination) or no therapy at all which could be changed during the treatment phase.’ | Yes |
| Harrison,  2017 | MAJIC-ET | The choice of second-line treatment in the control arm is at the Investigators discretion. This can be any active agent (non-investigational) such as continuing HC, anagrelide, interferon (any formulation), busulfan, 32P etc alone or in combination but not solely venesection or supportive care | Yes |
| Mesa,  2017 | RELIEF | Hydroxycarbamide | N/A |
| Mesa,  2017 | PERSIST-1 | ‘BAT consisted of any physician-selected treatment, excluding JAK2 inhibitors, and could also include no treatment (ie, watchful waiting) or symptom-directed treatment.’ | No |
| Kiladjian**,**  2020 | RESPONSE | - Hydroxyurea - IFN/PEG-IFN - Pipobroman - Anagrelide - Lenalidomide - Pomalidomide - Observation only | No |
| Passamonti,  2022 | RESPONSE-2 | Best Available Therapy as selected by the investigator from: Hydroxyurea, Pegylated-Interferon (IFN/PEG-IFN), pipobroman, anagrelide, IMIDs, or observation | No |
| Alvarez-Larrán,  2022 | - | BAT consisted of hydroxyurea (in 60% of the patients), interferon (4%), anagrelide (9%), busulfan (15%), melphalan (2%), radioactive phosphorus (1%), other treatments (2%), and no medication (8%) | N/A |
| Verstovsek,  2023 | MOMENTUM | Danazol | N/A |
| Harrison, 2023 | MAJIC-PV | The choice of second-line treatment in the control arm is at the Investigators discretion. This can be any active agent (non-investigational) such as continuing HC, anagrelide, interferon (any formulation), busulfan, 32P etc alone or in combination but not solely venesection or supportive care | Yes |

Abbreviations: BAT: Best available therapy

**Supplementary Figure S1: Version 2 of the Cochrane tool for assessing risk of bias in randomised trials**

**
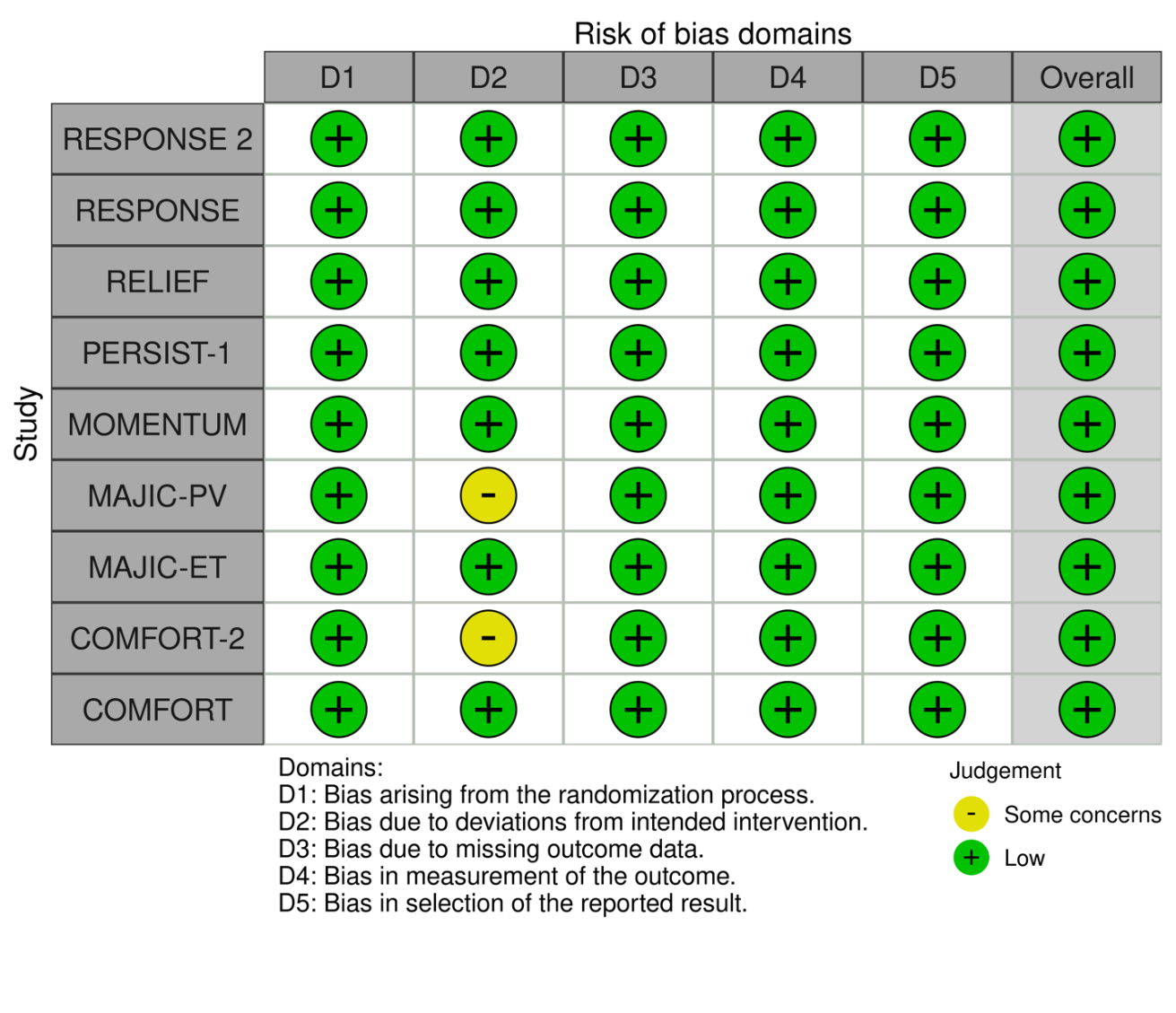
**

*Generated using RoB 2 tool,*

*Sterne JAC, Savović J, Page MJ, et al. RoB 2: a revised tool for assessing risk of bias in randomised trials. BMJ. 2019;366:l4898. Published 2019 Aug 28. doi:10.1136/bmj.l4898*

**Supplementary Figure S2: Risk Of Bias In Non-randomised Studies - of Interventions**

**
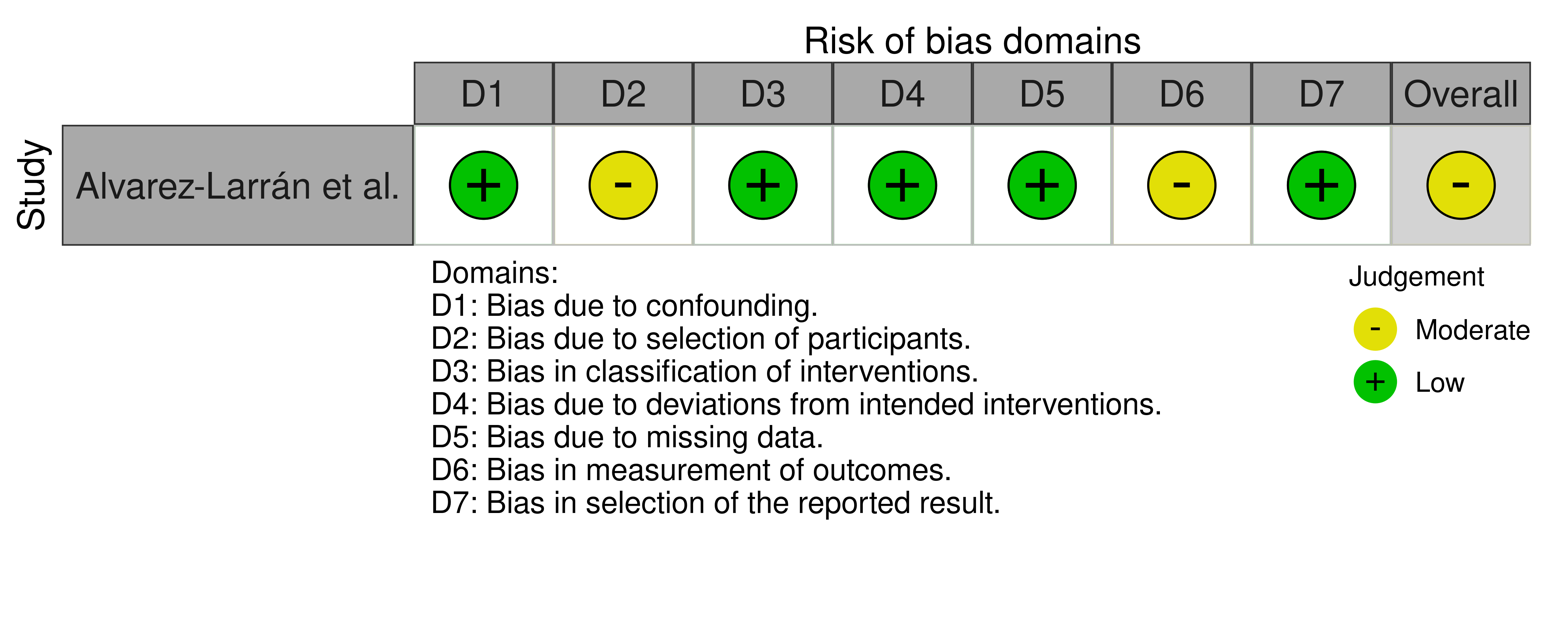
**

*Generated using ROBINS-I tool*

*Sterne JA, Hernán MA, Reeves BC, et al. ROBINS-I: a tool for assessing risk of bias in non-randomised studies of interventions. BMJ. 2016;355:i4919. Published 2016 Oct 12. doi:10.1136/bmj.i4919*
